# Validation of an IGF-CTP scoring system for assessing hepatic reserve in egyptian patients with hepatocellular carcinoma

**DOI:** 10.18632/oncotarget.4176

**Published:** 2015-05-19

**Authors:** Reham Abdel-Wahab, Samir Shehata, Manal M. Hassan, Lianchun Xiao, Ju-Seog Lee, Sheree Cheung, Hoda H. Essa, Hesham M. Hassabo, Ahmed S. Shalaby, Eman Mosad, Kanwal Raghav, Asif Rashid, Robert A. Wolff, Jeffrey S. Morris, Hesham M. Amin, Ahmed O. Kaseb

**Affiliations:** ^1^ Department of Gastrointestinal Medical Oncology, The University of Texas MD Anderson Cancer Center, Houston, Texas, USA; ^2^ Department of Biostatistics, The University of Texas MD Anderson Cancer Center, Houston, Texas, USA; ^3^ Department of Systems Biology, The University of Texas MD Anderson Cancer Center, Houston, Texas, USA; ^4^ Department of Pathology, The University of Texas MD Anderson Cancer Center, Houston, Texas, USA; ^5^ Department of Hematopathology, The University of Texas MD Anderson Cancer Center, Houston, Texas, USA; ^6^ Department of Clinical Oncology, Assiut University, Egypt; ^7^ Department of Pathology, Assiut University, Egypt; ^8^ Graduate School of Biomedical Sciences, Houston, Texas, USA

**Keywords:** IGF-1, Child-Pugh, validation, liver reserve, hepatocellular carcinoma

## Abstract

**Background:**

The Child-Turcotte-Pugh score (CTP) is the standard tool for hepatic reserve assessment in hepatocellular carcinoma (HCC). Recently, we reported that integrating plasma insulin-like growth factor-1 (IGF-1) level into the CTP score was associated with better patient risk stratification in two U.S. independent cohorts. Our current study aimed to validate the IGF-CTP score in patients who have different demographics and risk factors.

**Patients and Methods:**

We prospectively recruited 100 Egyptian patients and calculated their IGF-CTP score compared to CTP score. C-index was used to compare the prognostic significance of the two scoring systems. Finally, we compared our results with our U.S. cohorts published data.

**Results:**

IGF-CTP score showed significant better patient stratification compared to CTP score in the international validation cohort. Among CTP class A patients, who usually considered for active treatment and clinical trial enrollment, 32.5% were reclassified as IGF-CTP class B with significantly shorter OS than patients reclassified as class A with hazard ratio [HR] = 6.15, 95% confidence interval [CI] = 2.18-17.37.

**Conclusion:**

IGF-CTP score showed significantly better patient stratification and survival prediction not only in the U.S. population but also in international validation population, who had different demographics and HCC risk factors.

## INTRODUCTION

While several hepatocellular carcinoma (HCC) prognostic systems rely on the Child-Turcotte-Pugh (CTP) score to assess the underlying chronic liver disease (CLD) and to predict treatment outcome and overall survival (OS).[1] The original CTP scoring system was modified in 1973 to replace nutritional status with an objective variable, prothrombin time, and began to be used for assessment of life expectancy after ligation of bleeding esophageal varices in cirrhotic patients.[2] Patients classified according to CTP score into A, B, or C. Several trials have concluded that patients with CTP classes B and C have marked deterioration in hepatic function compared to those with class A. Therefore, typically, only patients with class A are eligible for active treatment.[3, 4]

The CTP score is still the standard tool for assessing the degree of hepatic reserve and predicting OS among cirrhotic and HCC patients.[1, 3, 4] However, the CTP scoring system includes two subjective variables (ascites and encephalopathy), which are difficult to assess as both may be influenced by medications, nutritional status and comorbidities.[5-10] These limitations raise the need for a more objective scoring system for better assessment of prognosis, prediction of survival, and decision-making about suitable treatment.[11, 12]

Our previous studies[13, 14] showed that the baseline plasma level of insulin-like growth factor-1 (IGF-1) was significantly associated with degree of CLD, HCC characteristics and patients’ OS duration. We therefore proposed substituting IGF-1 level for the two subjective parameters of the CTP score, creating the IGF-CTP (Kaseb-Morris) score. Recently, we found that the IGF-CTP score was significantly associated with better patient stratification compared to the CTP score in both U.S. training and validation cohorts.[15] The aims of the current study were to prospectively validate the IGF-CTP score in an independent cohort of 100 Egyptian patients and compare the results with the two previous cohorts. This study represents the first international validation of our recently reported IGF-CTP score, in a cohort of patients with different demographics, geographical location, and HCC risk factors.

## RESULTS

### Patient characteristics

*MD Anderson training cohort (USA)*: 420 HCC patients were enrolled between January 2000 and May 2008. Among those, 310 (73.8%) had available plasma samples.

*MD Anderson prospective validation cohort (USA)*: 197 HCC patients were enrolled between June 2008 and September 2011. Among those, 155 (78.7%) had available plasma samples.

*Assiut prospective validation cohort (Egypt)*: 100 HCC patients were enrolled between April 2012 and September 2013, and all had available plasma samples taken on the day of study enrollment in the clinic.

### Comparison between the Egyptian validation cohort and previous cohorts

The median follow-up times were 8.6 months (95% CI=6.8 to 14.5 months) for the Egyptian validation cohort, compared to 43.3 months (95% CI=41 to 53.5 months) for the training cohort and 16.5 months (95% CI=9.67 to 24.1 months) for the MD Anderson validation cohort.

Table 1 highlights the statistically significant variations in patient characteristics between the three cohort groups. The mean age for Egyptian patients was 56.7 years (standard deviation (SD), ± 8.7 years), compared to 62.6 years (SD ±11.8 years) and 63.2 years (SD ±10.8 years) for the training and validation cohorts, respectively. Notably, 66% of the Egyptian patients were 60 years or younger at the time of diagnosis. Among the Egyptian patients, 83% were male, with a higher male:female ratio (4.9:1) compared to the other cohorts. The Egyptian patients had higher α-FP, ALT values, lower platelet and serum sodium (Na) level and more cirrhosis, vascular invasion, advanced CTP score, advanced BCLC stage, and systemic therapy usage compared with the other cohorts. Also, the mean coefficient for IGF-1 was 0.7in the training cohort, 1.38 in MDACC validation cohort, and 0.42 in the Egyptian validation cohort.

**Table 1:** Baseline characteristics of patients in the training and validation cohorts

Patient characteristic	Parameter	Training cohort N=310 (%)*	First validation cohort N=155 (%)*	Second validation cohort N=100 (%)	*P* value
Age (years)	≤60	136 (43.9%)	67 (43.2%)	66 (66%)	.0003
	>60	174 (56.1%)	88 (56.8%)	34 (34%)	
Sex	Male:female ratio	2.4:1	2.7:1	4.9:1	
	Male	218 (70.3%)	113 (72.9%)	83 (83%)	.04
	Female	92 (29.7%)	42 (27.1%)	17 (17%)	
Viral hepatitis	HCV, HBV, or both	139 (44.8%)	78 (50.3%)	100 (100%)	< .0001
	None	171 (55.2%)	77 (49.7%)	0	
Serum α-FP (ng/mL)	<400	207 (66.8%)	99 (63.9%)	48 (48%)	.001
	≥400	97 (31.3%)	56 (36.1%)	52 (52%)	
	Missing	6	0	0	
Tumor differentiation	Well	122 (39.4%)	49 (31.6%)	4 (4%)	< .0001
	Moderate	100 (32.3%)	42 (27.1%)	15 (15%)	
	Poor	52 (16.7%)	38 (24.5%)	5 (5%)	
	Missing (no biopsy)	36 (11.6%)	26 (16.8%)	76 (76%)	
Tumor nodularity	Uninodular	108 (34.8%)	47 (30.3%)	33 (33%)	.5
	Multinodular	192 (61.9%)	108 (69.7%)	64 (64%)	
	Missing	10	0	0	
% of liver involvement	≤50%	203 (65.5%)	113 (72.9%)	66 (66%)	.3
	>50%	103 (33.2%)	41 (26.5%)	27 (27%)	
	Missing	4	1	7	
Vascular invasion	Yes	88 (28.4%)	54 (34.8%)	49 (49%)	.0006
	No	221 (71.3%)	100 (64.5%)	50 (50%)	
	Missing	1	1	1	
Lymph node metastasis	Yes	126 (40.6%)	90 (58.1%)	47 (47%)	.003
	No	181 (58.4%)	65 (41.9%)	53 (53%)	
	Missing	3	0	0	
Extrahepatic metastasis	No	242 (78.1%)	70 (45.2%)	76 (76%)	<.0001
	Yes	66 (21.3%)	85 (54.8%)	24 (24%)	
	Missing	2	0	0	
ALT (U/L)	≤40	137 (44.2%)	67 (43.2%)	29 (29%)	0.04
	>40	171 (54.8%)	88 (56.8%)	67 (67%)	
	Missing	2	0	4	
AST (U/L)	≤45	93 (30%)	26 (16.8%)	16 (16%)	.0001
	>45	192 (61.9%)	129 (83.2%)	80 (80%)	
	Missing	25	0	4	
Platelet (mcl)	≤100	34 (14.3%)	32 (20.6%)	39 (39%)	<.0001
	>100	204 (85.7%)	123 (79.4%)	61 (61%)	
	Missing	72	0	0	
Na (mEq/L)	≤130	10 (3.3%)	5 (3.2%)	25 (29.4%)	<.0001
	>130	295 (96.7%)	149 (96.8%)	60 (70.6%)	
	Missing	5	1	15	
Cirrhosis	No	116 (37.4%)	52 (36.4%)	13 (13%)	< .0001
	Yes	194 (62.6%)	93 (63.6%)	87 (87%)	
CTP score	A	221 (71.2%)	126 (81.3%)	40 (40%)	< .0001
	B	79 (25.5%)	25 (16.1%)	32 (32%)	
	C	8 (2.6%)	4 (2.6%)	28 (28%)	
	Missing	2	0	0	
BCLC stage	0	20 (6.5%)	2 (1.3%)	0	<.0001
	A	27 (8.7%)	13 (8.4%)	1 (1%)	
	B	30 (9.7%)	17 (11%)	8 (8%)	
	C	196 (63.2%)	119 (76.8%)	60 (60%)	
	D	23 (7.4%)	4 (2.5%)	31 (31%)	
	Missing	14	0	0	
Treatment history	Systemic ± Local	143 (46.1%)	89 (57.4%)	64 (64%)	<.0001
	Local therapy only (TACE/RFA)	29 (9.4%)	40 (25.8%)	12 (12%)	
	Surgery ± Local	94 (30.3%)	15 (9.7%)	1 (1%)	
	Best supportive care	44 (14.2%)	11 (7.1%)	23 (23%)	

**Abbreviations:** HCV, hepatitis C virus; HBV, hepatitis B virus; α-FP, α-fetoprotein; ALT, alanine transaminase; AST, aspartate transaminase; CTP, Child-Turcotte-Pugh; BCLC, Barcelona Clinic Liver Cancer; TACE, trans-arterial chemoembolization; RFA, radiofrequency ablation.

* Data reprinted from reference 15 with permission.

### Comparison of OS duration and prognostic accuracy by CTP score vs. IGF-CTP score

The median OS duration in the Egyptian validation cohort was 8.05 months (95% CI=6.9 to 9.2 months), compared to 13.2 months (95% CI=11.4 to 16.6 months) in the training cohort and 15.7 months (95% CI=12.2 to 19.9 months) in the MD Anderson validation cohort. Table 2 summarizes OS according to the plasma IGF-1 levels. Patients with a high IGF-1 level (>50 ng/ml) had significantly longer OS than those with intermediate and low IGF-1 levels, in the Egyptian validation cohort (*P* < .0001); the same was true for both the training and MD Anderson validation cohorts (*P* < .001).

**Table 2:** Log-rank and Cox model results for overall survival of the training and validation cohorts based on IGF-1 level

		Training cohort (N=310) *				First validation cohort (N=155) *				Second validation cohort (N=100)			
Overall survival by IGF-1 level	IGF-1 level (ng/mL)	No.(%)	E	Median OS, months (95% CI)	*P* value	No.(%)	E	Median OS, months (95% CI)	*P* value	No. (%)	E	Median OS, months (95% CI)	*P* value
All patients		310	238	13.22(11.4 to 16.6)		155	71	15.7(12.2 to 19.9)		100	100	8.05(6.9 to 9.2)	
	>50	133 (42.9%)	92	22.6(15.1 to 28.8)	<.001	61 (39.4%)	24	23.7(18.4 to NA)	<.001	44 (44%)	33	10.05(9.9 to 10.2)	<.0001
	26-50	109 (35.2%)	85	13.6(8.5 to 19.3)		17 (10.9%)	5	9.5(7.6 to NA)		20 (20%)	18	9.04(5.9 to 12.2)	
	<26	68 (21.9%)	61	5.0(4.01 to 11.9)		77 (49.8%)	42	9.4(6.4 to 14.74)		36 (36%)	33	8.05(6.9 to 9.2)	
													
**Hazard ratios by IGF-1 level**				**HR (95% CI)**	***P* value**			**HR (95% CI)**	***P* value**			**HR (95% CI)**	***P* value**
	>50	—	—	1.00 (reference)				1.00 (reference)		—	—	1.00 (reference)	
	26-50	—	—	1.45(1.08 to 1.95)	.01			1.26(0.48 to 3.33)	.64	—	—	1.4(0.81 to 2.58)	.22
	<26	—	—	2.5(1.8 to 3.47)	<.001			2.81(1.68 to 4.68)	<.001	—	—	5.16(2.99 to 8.9)	<.0001
		—	—							—	—		
	26-50	—	—	1.00 (reference)	—			1.00 (reference)		—	—	1.00 (reference)	
	<26	—	—	1.72(1.24 to 2.4)	.001			2.28(0.9 to 5.76)	.08	—	—	3.(1.76 to 6.6)	<.0001

**Abbreviations:** N, number; E, event (death); OS, overall survival; CI, confidence interval; IGF-1, insulin-like growth factor-1; NA, not available

* Data reprinted from reference 15 with permission.

Among all three cohorts, patients with low IGF-1 levels had statistically significantly shorter OS compared to patients with high IGF-1 levels. Patients with an intermediate IGF-1 level had shorter OS compared to patients with a high IGF-1 level, but this difference was not statistically significant in either validation cohort. Furthermore, patients with a low IGF-1 level had shorter OS compared to patients with an intermediate IGF-1 level in the Egyptian validation cohort (*P* < 0.0001), as well as in the training cohort (*P* = 0.001). In the MD Anderson validation cohort, patients with a low IGF-1 level had a worse prognosis than that of patients with an intermediate IGF-1 level, but this difference was not statistically significant (HR=2.28, *P* = 0.082).

Table 3 shows that median OS was statistically significantly correlated with different risk groups in both scoring systems. Concordance index (C-index) yields values ranging from 0 (no discrimination) to 1 (perfect separation) to compare different prognostic systems. It has been previously used in the landmark paper that reported MELD score to determine organ allocation priorities.[16] C-index analysis (Table 4) demonstrated that the differences between C-indices were not large, but the prognostic stratification provided by IGF-CTP score was statistically significant compared to CTP score in the Egyptian validation cohort (*P* = 0.003) as well as in the training cohort (*P* = 0.003) and MD Anderson validation cohort (*P* = 0.005). Similarly, MELD score study reported a C-index of 0.83 for MELD score as compared to 0.76 for the CTP. Notably, the difference between C-indices was not large (=0.07), however, it was statistically significant, *P* < 0.001. This is because the C-index computes the prognostic score ability to predict OS for all patients in the cohort, including those whose CTP and IGF-CTP scores were different and those whose scores were the same. To better understand this improvement, we focused on the patients who were classified into different risk groups by the two scoring systems.

**Table 3:** Log-rank and Cox model results for overall survival of the training and validation cohorts by Child-Turcotte-Pugh (CTP) and IGF-CTP class

		Training cohort (N = 310) *				First validation cohort (N = 155) *				Second validation cohort (N = 100)			
Scoring system	Class	No	E	Median OS,	*P* value	No	E	Median OS,	*P* value	No	E	Median OS,	*P* value
				months (95% CI)				months (95%CI)				months (95%CI)	
IGF-CTP	A (4-5)	186	132	20.5 (15.0 to 26.7)	<.001	70	25	25.9 (18.4 to NA)	<.001	35	28	11.96 (9.8 to 14.2)	<.0001
	B (6-7)	87	72	11.5 (8.3 to 16.3)		70	38	9.5 (7.7 to 16.2)		37	32	8.02 (6.03 to 10)	
	C (>7)	26	25	2.5 (2.2 to 5.7)		15	8	5.1 (2.1 to NA)		28	24	6.31 (5.4 to 7.2)	
CTP	A (5-6)	221	163	17.1 (12.9 to 21.4)	<.001	126	56	16.9 (13.2 to 25.1)	<.001	40	31	11.01 (9.9 to 12.1)	<.0001
	B (7-9)	79	67	6.5 (4.8 to 13.1)		25	12	8.8 (7.1 to NA)		32	26	7.52 (6.7 to 8.3)	
	C (>10)	8	7	2.6 (0.7 to NA)		4	3	2.1 (0.5 to NA)		28	27	5.65 (4.4 to 6.9)	
													
				**HR (95% CI)**	***P* value**			**HR (95% CI)**	***P* value**			**HR (95% CI)**	***P* value**
IGF-CTP	A	—	—	1.00 (reference)				1.00 (reference)		—	—	1.00 (reference)	
	B	—	—	1.61 (1.20 to 2.15)	.001			2.67 (1.61 to 4.44)	< .001	—	—	2.67 (1.5 to 4.6)	<.0001
	C	—	—	6.34 (4.03 to 9.97)	<.001			7.70 (3.34 to 17.74)	< .001	—	—	12.94 (6.4 to 26.2)	<.0001
		—	—							—	—		
	B	—	—	1.00 (reference)				1.00 (reference)		—	—	1.00 (reference)	
	C	—	—	3.94 (2.47 to 6.31)	<.001			2.88 (1.31 to 6.34)	.008	—	—	4.05 (2.1 to 7.7)	<.0001
CTP	A	—	—	1.00 (reference)				1.00 (reference)		—	—	1.00 (reference)	
	B	—	—	1.57 (1.18 to 2.09)	.002			1.94 (1.03 to 3.06)	.04	—	—	5.24 (2.8 to 9.8)	<.0001
	C	—	—	4.63 (2.15 to 9.94)	<.001			17.12 (4.94 to 59.31)	< .001	—	—	14.7 (7.6 to 28.7)	<.0001
		—	—							—	—		
	B	—	—	1.00 (reference)				1.00 (reference)		—	—	1.00 (reference)	
	C	—	—	2.94 (1.31 to 6.45)	.007			8.84 (2.35 to 33.24)	.001	—	—	2.56 (1.5 to 4.5)	.001

**Abbreviations:** N, number; E, event (death); OS, overall survival; CI, confidence interval; NA, not applicable; CTP, Child-Turcotte-Pugh; IGF, insulin-like growth factor-1.

*Parameters from 11 and two patients were not available for calculation of their IGF-CTP and CTP scores, respectively.

* Data reprinted from reference 15 with permission.

**Table 4:** Ranking of scoring systems by C-index

Patient cohort	Scoring system	C-index (95% CI)	*P* value
Training cohort N=310*	IGF-CTP	0.608 (0.606 to 0.610)	.003
	CTP	0.573 (0.571 to 0.575)	
First validation cohort N=155*	IGF-CTP	0.672 (0.666 to 0.677)	.005
	CTP	0.579 (0.576 to 0.583)	
**Second validation cohort N=100**	IGF-CTP	0.641 (0.637 to 0.645)	.003
	CTP	0.597 (0.596 to 0.601)	

**Abbreviations:** IGF, insulin-like growth factor-1; CTP, Child-Turcotte-Pugh; N, number; CI, confidence interval.

* Data reprinted from reference 15 with permission.

### Reassignment of patients from CTP classes to different IGF-CTP classes

**Figure 1:**
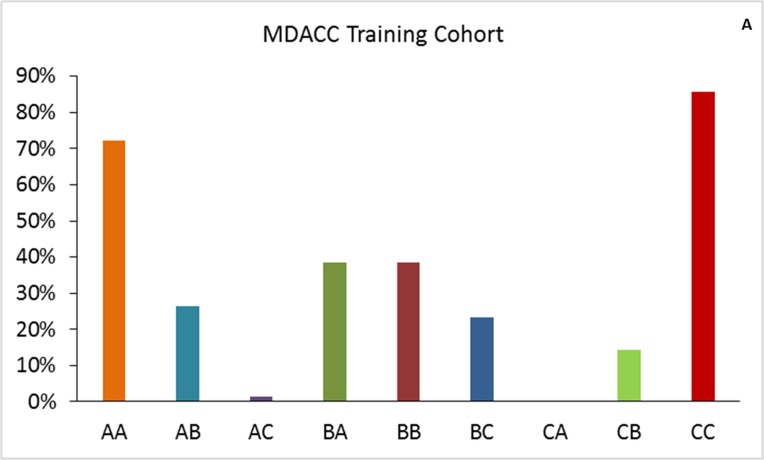

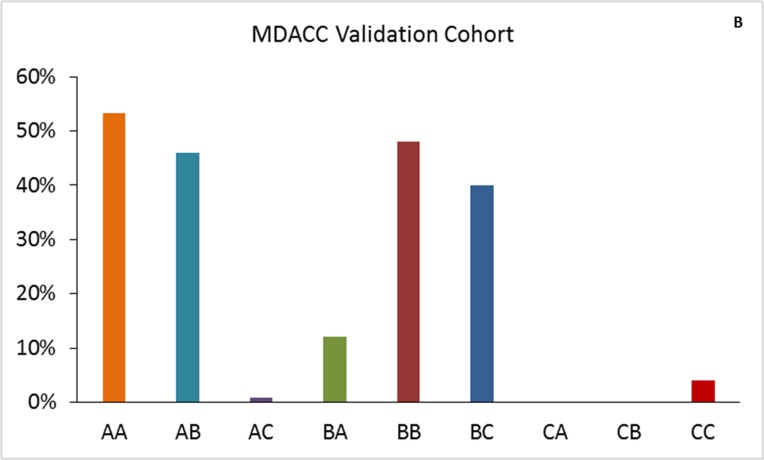

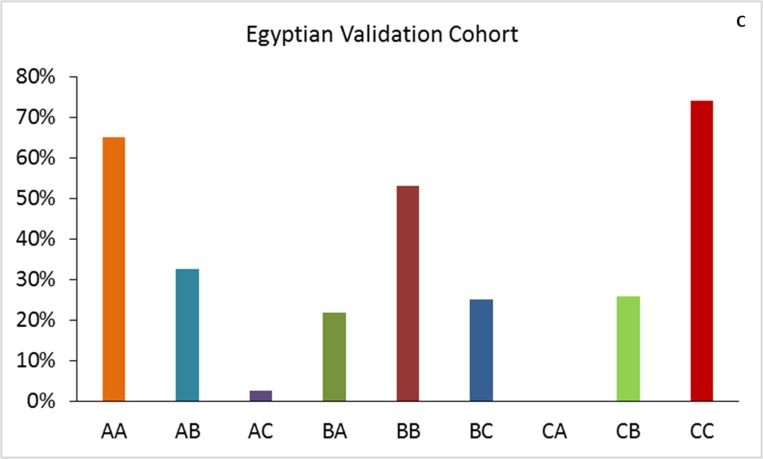
Patient distribution in the training and validation cohorts for IGF-CTP score class by CTP score class The first letter for each group represents the CTP class; the second letter, the IGF-CTP class (e.g., group AB represents patients classified as CTP class A and IGF-CTP class B).

**Figure 2:**
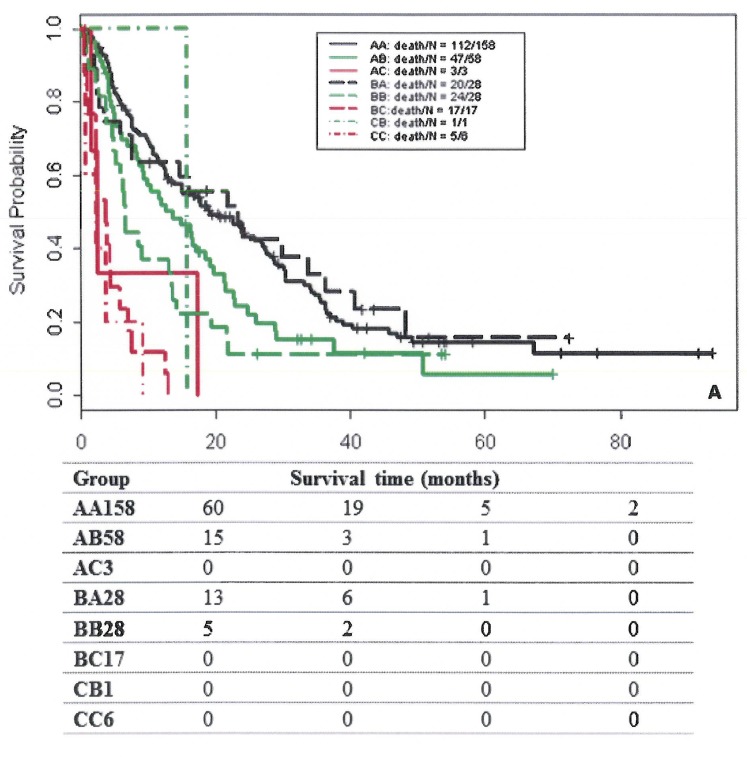

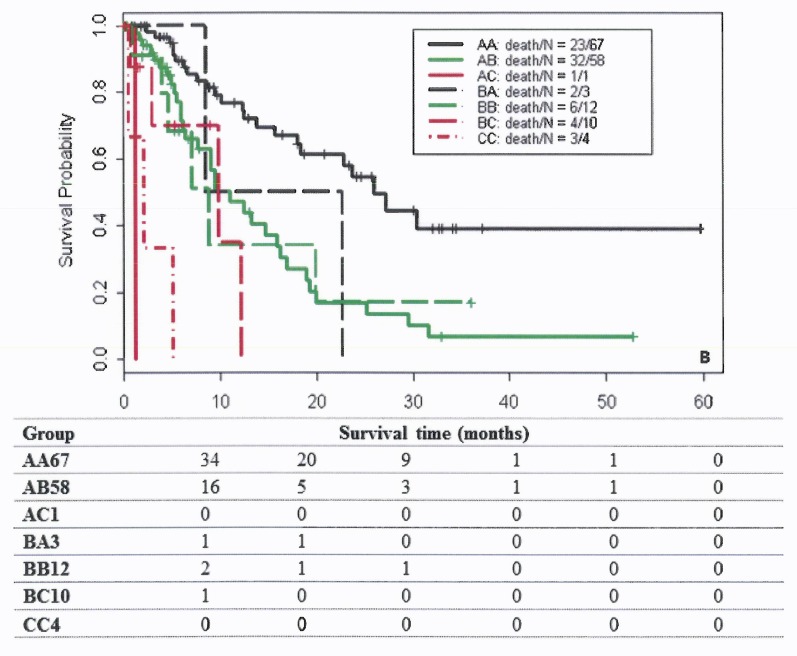

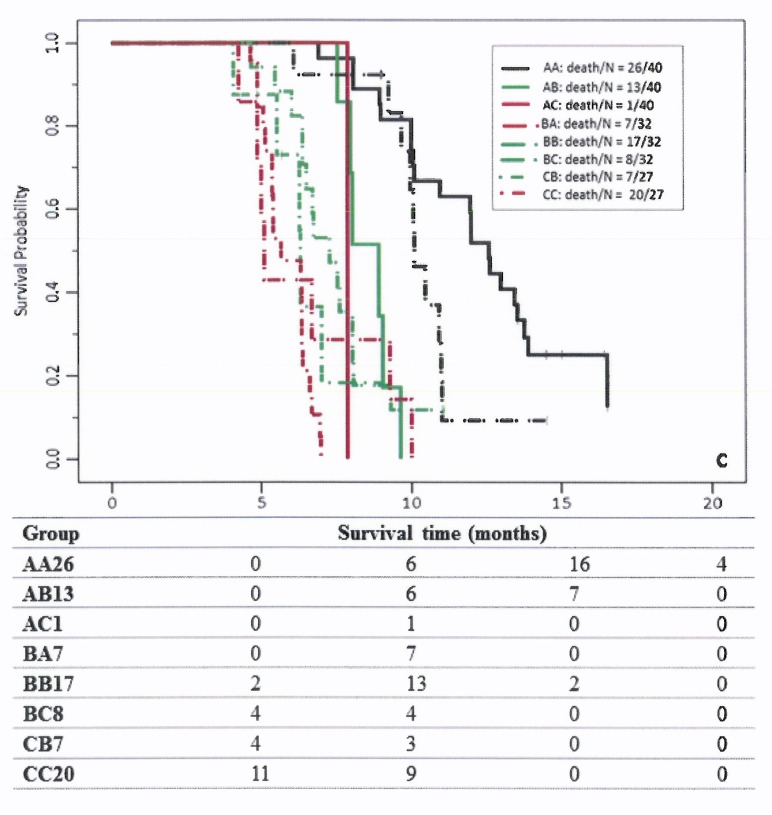
Survival curves of patients classified by group in the training cohort (**A**), MDACC validation cohort (**B**), and Egyptian validation cohort (**C**). The first letter for each group represents the CTP class; the second letter, the IGF-CTP class (e.g., group AB represents patients classified as CTP class A and IGF-CTP class B).

We found that 63% of patients in the Egyptian cohort were classified in the same risk groups by both scoring systems, compared to 61.9% in the training cohort and 53.5% in the MD Anderson validation cohort (Figure 1). Comparison of OS durations (Table 5) and Kaplan-Meier survival curves (Figure 2) showed a significant difference when patients were stratified with the IGF-CTP score compared to the original CTP score.

**Table 5:** Overall survival of the training and validation cohorts by whether CTP class was reclassified in the IGF-CTP scoring system

	Training cohort (N = 310) *				First validation cohort (N= 155) *				Second validation cohort (N = 100)			
	N	E	Median OS, months (95% CI)	*P* value	N	E	Median OS, months (95% CI)	*P* value	N	E	Median OS, months (95% CI)	*P* value
Original A to new A (AA)	158	112	19.3 (14.9 to 27.0)	<0.001^¶^	67	23	25.9 (18.4 to NA)	<0.001^¶^	26	20	12.58 (11.7 to 13.4)	<0.0001^¶^
Original A to new B (AB)	58	47	13.6 (9.1 to 19.7)		58	32	11.0 (7.7 to 16.9)		13	10	8.9 (7.8 to 10)	
Original A to new C (AC)	3	3	2.3 (1.5 to NA)		1	1	1.2 (NA to NA)		1	1	7.85 (NA to NA)	
Original B to new A (BA)	28	20	23.5 (7.6 to 40.6)		3	2	15.6 (8.5 to NA)		7	7	10.09 (9.5 to 10.7)	
Original B to new B (BB)	28	24	6.5 (5.1 to 13.6)		12	6	8.8 (4.6 to NA )		17	15	7.26 (6.2 to 8.4)	
Original B to new C (BC)	17	17	3.7 (2.2 to 6.9)		10	4	9.8 (2.9 to NA)		8	5	5.09 (4.8 to 5.3)	
Original C to new A (CA)	0	-	-		0	-	-		0	-	-	
Original C to new B (CB)	1	1	15.9 (NA to NA)		0	-	-		7	7	8.05 (6.9 to 9.2)	
Original C to new C (CC)	6	5	2.3 (0.7 to NA)		4	3	2.1 (0.5 to NA)		20	19	5.65 (4.6 to 6.7)	
												
			**HR (95% CI)**	***P* value**			**HR (95% CI)**	***P* value**			**HR (95% CI)**	***P* value**
AA			1.00 (reference)				1.00 (reference)				1.00 (reference)	
AB			1.45 (1.03 to 2.04)	.03			2.83 (1.65 to 4.85)	< .001			6.15 (2.18 to 17.37)	.001
AC			4.05 (1.28 to 12.83)	.02			NA	NA			10.11 (1.25 to 81.75)	.03
BA			0.95 (0.59 to 1.53)	.84			2.63 (0.62 to 11.23)	.19			2.02 (0.91 to 4.46)	.08
BB			2.0 (1.28 to 3.11)	.002			2.87 (1.17 to 7.08)	.02			6.83 (3.15 to 14.78)	<.0001
BC			6.45 (3.78 to 11.01)	<.001			4.66 (1.57 to 13.86)	.006			11.93 (4.55 to 31.3)	<.0001
CB			1.69 (0.24 to 112.16)	.60			NA	NA			14.25 (4.67 to 43.48)	<.0001
CC			8.94 (3.59 to 22.23)	<.001			32.39 (8.86 to 118.49)	< .001			37.36 (15.05 to 92.74)	<.0001
												
BB			1.00 (reference)				1.00 (reference)				1.00 (reference)	
AB			0.73 (0.44 to 1.19)	.20			0.98 (0.41 to 2.36)	.97			0.94 (0.35 to 2.49)	.89
AC			2.03 (0.61 to 6.77)	.25			NA	NA			1.46 (0.19 to 11.34)	.72
BA			0.48 (0.26 to 0.87)	.03			0.92 (0.18 to 4.57)	.91			0.37 (0.16 to 0.83)	.02
BC			3.23 (1.72 to 6.09)	<.001			1.62 (0.45 to 5.86)	.46			1.891 (0.75 to 4.79)	.18
CB			0.85 (0.11 to 6.28)	.87			NA	NA			2.23 (0.78 to 6.41)	.14
CC			4.48 (1.69 to 11.8)	.003			11.27 (2.64 to 48.14)	.001			5.41 (2.42 to 12.1)	<.0001
												
CC			1.00 (reference)				1.00 (reference)				1.00 (reference)	
AB			0.11 (0.05 to 0.28)	<.001			0.09 (0.02 to 0.31)	< .001			0.18 (0.06 to 0.52)	.002
AC			0.45 (0.11 to 1.91)	.28			NA	NA			0.3 (0.04 to 2.35)	.25
BA			0.11 (0.04 to 0.29)	<.001			0.08 (0.01 to 0.52)	.008			0.03 (0.007 to 0.11)	<.0001
BC			0.72 (0.27 to 1.96)	.52			0.14 (0.03 to 0.68)	.01			0.27 (0.09 to 0.85)	.03
CB			0.19 (0.02 to 1.63)	.13			NA	NA			0.42 (0.15 to 1.19)	.103

**Abbreviations:** IGF, insulin-like growth factor-1; CTP, Child-Turcotte-Pugh; N, number; E, event(death); NA, not applicable; OS, overall survival.

¶ *P* value compares across all groups.

* Data reprinted from reference 15 with permission

In the Egyptian validation cohort, 26/40 (65%) of the patients with CTP class A were classified as IGF-CTP-A (AA), and they had an OS duration of 12.58 months (95% CI=11.7 to 13.4 months). While, 13/40 (32.5%) were reclassified as IGF-CTP-B (AB), and they had significantly worse OS of 8.9 months (95% CI=7.8 to 10 months) compared to group AA. Only one patient was reclassified as IGF-CTP-C (AC). Patients reclassified from CTP-A to IGF-CTP-B (AB) or IGF-CTP-C (AC) had worse prognosis compared to patients who were classified as AA (HR=6.15; *P* = 0.001 and HR=10.11; *P* = .03, respectively).

Moreover, patients who were reclassified from CTP-B to IGF-CTP-A (BA) had better prognosis compared to patients who were classified as BB (HR=0.37; *P* = 0.02). Finally, the majority of CTP-C patients remained in class C according to the IGF-CTP scoring system. Only 7/27 (25.9%) were reclassified as IGF-CTP-B, and their OS duration was 8.05 months (95% CI=6.9 to 9.2 months); this duration did not differ significantly when compared to patients who were classified as CC (OS=5.65 months; HR=0.42; 95% CI=0.15 to 1.19; *P* = 0.103).

## DISCUSSION

In this study in an Egyptian population, we have validated the plasma level of IGF-1 as a surrogate marker for functional liver reserve and the value of its integration into the CTP scoring system in place of encephalopathy and ascites. We conclude that the IGF-CTP scoring system is associated with significantly better HCC patient stratification and survival prediction not only in the U.S. populations originally tested but also in our validation population, who had different demographics, geographical location, and HCC risk factors than those previously reported.

Notably, HCC is a heterogeneous disease because of variation in the underlying cause, with subsequent variation in the mechanism of development. In the USA, 40% of HCC cases are attributed to chronic HCV infection alone. In Egypt, the incidence of HCC has been soaring over the past two decades, and it is now the most common cancer in Egyptian men, related to HCV in up to 94% of cases.[17-19]

Furthermore, there is variation in HCV genotypes: up to six types and more than 80 subtypes exist.[20] In the United States, 70% of cases have HCV genotype 1a/1b, 15-20% genotype 2, about 10% genotype 3, 1% genotype 4 and less than 1% genotypes 5 and 6.[21] In contrast, in Egypt, the prevalence of genotype 4a/4b is 91%; among those, 63% are genotype 4a.[22-24] Previous studies[25, 26] showed a higher rate of recurrent advanced fibrosis following liver transplantation among patients with recurrent HCV genotype 4 infection compared to other genotypes. This association of liver fibrosis and HCV genotype 4 infection could explain the higher rate of advanced cirrhosis in Egyptian HCC patients.

In our study, 66% of the Egyptian patients were diagnosed at a younger age, a higher percentage compared to U.S. patients. The Egyptian patients had a statistically significant higher α-FP and ALT level with more incidence of cirrhosis, vascular invasion, advanced CTP score, advanced BCLC stage, and systemic therapy usage. These findings suggest a more aggressive nature of the disease among Egyptian patients and subsequently their lower hepatic reserve capacity. Therefore, developing a more sensitive tool to assess hepatic reserve is critical to this patient population management.

Furthermore, while surgical resection and liver transplantation are the only curative treatment for HCC, unfortunately, most patients are not surgical candidates due to either advanced disease at the time of presentation and/or advanced underlying CLD. Therefore, assessing hepatic reserve in HCC is expected to have a great impact on treatment decision and predicted patients’ overall survival in addition to stratification and recruitment of patients for clinical trials and estimation of patients’ OS.[27].

Notably, sorafenib is the only systemic treatment approved for treatment of CTP-A HCC patients.[28, 29] However, its high cost adds a significant burden to health care system budgets. Since Egypt, which is considered a lower-middle-income country by the World Bank[30] and the International Monetary Fund,[31] has limited resources, it is thus particularly important to identify which HCC patients will benefit from sorafenib as distinct from those in whom sorafenib will pose higher rate of adverse events and lower survival benefit. In this context, applying a simple, noninvasive, low cost marker for liver reserve assessment will be helpful.[32].

CTP is the standard tool used to assess underlying liver reserve within major HCC staging systems such as Cancer of the Liver Italian Program (CLIP), and BCLC. Several studies have concluded that only patients with CTP class A are likely to benefit from active treatment and enrollment into clinical trials, while patients with CTP class B or C disease have significantly decreased survival expectancy due to the deterioration in hepatic function compared to class A.[3, 4] When comparing the results of the new IGF-CTP score in this international validation cohort with those of our previous training and validation cohorts at MD Anderson, we found that in all three groups, some patients with CTP-A were reclassified into by IGF-CTP scoring system into classes B and C; these patients had poorer hepatic reserve and therefore shorter OS. Accurate selection of patients with CTP class A is extremely important in the clinical practice because these group of patients who are illegible for active treatment. On the contrary, some patients with CTP-B were reclassified as class A based on the new scoring system and had a longer OS but they prohibited from receiving active treatment based on the original CTP score that classify them as class B. Therefore, the new IGF-CTP score could led to more precise ability to select patients who will benefit from treatment (class A).

The major strength of our study is that this is the first international validation of the IGF-CTP score, and it is tested through an independent prospective validation cohort in patients with different demographics, geographical location, hepatitis status and other HCC risk factors from those of the original cohorts. In the Egyptian cohort, all patients had viral-induced HCC, compared with only 44.8% in the training cohort and 50.3% in the first validation cohort. Our findings help to identify the prognostic significance of this score in patients who have more advanced disease and marked impairment of the liver capacity both by HCC as a space-occupying lesion and by the underlying viral hepatitis, which is mainly induced by HCV genotype 4, with a higher degree of liver fibrosis. Another strength of this study is that since the majority of patient's had unresectable disease; the main pool of patients undergoing systemic and local therapies in routine practice and clinical trials; accurate assessment of hepatic reserve and subsequent prediction of patient's prognosis and survival in this group of patients is very important to identify who will get benefit from active therapy and potential enrollment in clinical trials.

“Our study has some limitations. First, the majority of patients had unresectable disease. However, predicting patient's prognosis and survival in this group of patients is very important to identify who will benefit from systemic therapy and potential enrollment in clinical trials. Second, we don't have complete data regarding alcohol intake. However, all cases were viral related and therefore cirrhosis in the Egyptian population was mainly related to hepatitis virus. Finally, A limitation of our study was that it didn't allow us to test the predictive value of the IGF-CTP score for the outcome of different treatment modalities. Therefore, future studies to evaluate the predictive ability of IGF score in systemic and local therapies are warranted”.

Despite the CTP score's limitations, it is still the only standard tool parameter for assessing underlying liver reserve capacity within the most clinically used staging systems as CLIP, CUPI, and BCLC. So, this will affect patient staging and subsequantly selecting the suitable treatment, predicting treatment outcome, and identifing patients who are eligible to be enrolled in clinical trials. In the current study, we validate the value of incorporating plasma IGF-1 level instead of encephalopathy and ascites as parameters of the CTP score to create a new scoring system, Kaseb-Morris IGF-CTP system.

The new IGF-CTP score significantly improved selection of patients who were candidates for treatment and prediction of survival outcome. Since the majority of HCC staging systems include CTP score in their parameters,1 future studies to assess the value of integrating IGF-CTP score instead of CTP score in these staging systems may lead to more accurate patient stratification and treatment selection.” After further validation in HCC patients with different demographics and risk factors, the new IGF-CTP score may help to improve prediction of survival outcomes and rate of therapy adverse events which may subsequently aid in better selection of patients who are candidates for active HCC treatment and in prediction of survival outcome. Finally, since the majority of HCC staging systems include CTP score in their parameters as a liver assessment tool,1 future studies to assess the value of integrating IGF-CTP score instead of CTP score in these staging systems may lead to more accurate patient stratification and treatment selection.

## PATIENTS AND METHODS

### Study design and study population

We recruited 100 HCC patients from Assiut University Hospital, Egypt, from April 2012 to September 2013. We compared our cohort with two previous cohorts: i) prospectively recruited HCC patients who presented at MD Anderson Cancer Center from January 2000 to May 2008 and were used as a training cohort, and ii) prospectively recruited HCC patients who presented at MD Anderson between June 2008 and September 2011 and were used as a validation cohort. This study was approved by the institutional review boards of MD Anderson and Assiut University Hospital. Written informed consent for study participation was obtained from each patient. For all three HCC cohorts, the study involved pathologically confirmed HCC or radiologically confirmed HCC based on American Association for the Study of Liver Diseases guidelines.[33] for patients enrolled after 2005 who did not have available biopsy samples.

Detailed data on patients’ demographics, risk factors, and clinicopathological features were collected. The clinical data included information about Eastern Cooperative Oncology Group (ECOG) performance status; levels of alpha-fetoprotein (α-FP), aspartate transaminase (AST), and alanine transaminase (ALT;presence of cirrhosis; tumor nodularity; volume of the liver occupied by the tumor; vascular invasion; lymph node metastasis; extrahepatic metastasis; CTP score; Barcelona Clinic Liver Cancer (BCLC) stage; and treatment history. We followed standard approach to calculate CTP score, by using clinical judgment and imaging studies to assess ascites and encephalopathy which is currently the standard approach in routine practice and clinical trials, therefore limited since it is subjective parameters and influenced by other factors

Blood samples for IGF-1 analysis were prospectively collected and analyzed for IGF-1 in our cohort. Our data were compared with the IGF-1 values in the training cohort, for which samples were prospectively collected and retrospectively analyzed, and in the validation cohort, for which samples were prospectively collected and analyzed.

### Baseline plasma IGF-1 level

Peripheral venous blood samples (3-5 mL) were collected, anticoagulated by ethylenediaminetetraacetic acid (EDTA), and centrifuged at 4°C for 15 minutes at 3000 RPM. Then the plasma samples were removed, aliquoted, and snap-frozen at −20°C until analyzed.

In both the training cohort and the Egyptian validation cohort, IGF-1 was tested by enzyme-linked immunosorbent assay (ELISA) according to the manufacturer's directions (Quantikine Human IGF-1 ELISA Kit; R&D Systems, Minneapolis, MN). In the MD Anderson validation cohort, plasma IGF-1 was tested at a Clinical Laboratory Improvement Amendments (CLIA)–certified facility that uses Luminex microsphere technology by Myriad Laboratories (Austin, Texas).

### IGF-CTP score

Patients’ IGF-CTP scores were calculated and class A, B, or C assigned based on their serum bilirubin level, serum albumin level, prothrombin time, and plasma IGF-1 level as described previously.[15]

### Statistical analysis

We used IBM SPSS Statistics for Windows, Version 21.0 (IBM Corp., Armonk, NY) for data management and statistical analysis. Univariate analysis was done using chi-square or Fisher's exact test for categorical variables and the Kruskal-Wallis test for continuous variables. The Kaplan-Meier method was used to estimate the median overall survival (OS), which was defined as the time interval between the date of the blood draw for IGF-1 measurement and the date of death or last follow-up. Also, the log-rank test was used to detect the statistical significance between the CTP score and the new IGF-CTP score subgroups. Then, we used the Cox proportional hazard model to calculate hazard ratios (HRs) and 95% confidence intervals (95% CIs) to identify independent prognostic factors for OS. Finally, we calculated the Harrell's C-index to compare the prognostic performance of both scores among the three cohorts. For all statistical analyses, a two-sided *P* value as a descriptive measure was considered statistically significant if less than 0.05.

## SUPPLEMENTARY MATERIAL TABLE


