# Supplementary material for: Validation of an IGF-CTP scoring system for assessing hepatic reserve in egyptian patients with hepatocellular carcinoma
Source: Oncotarget. 2015 May 19;6(25):21193–207. doi: 10.18632/oncotarget.4176 (PMC4673259; doi:10.18632/oncotarget.4176)
Supplement: Supplementary file 1 [file oncotarget-06-21193-s001.pdf]

Validation of an IGF-CTP scoring system for assessing hepatic reserve in egyptian patients with hepatocellular carcinoma

Supplementary Material

Supplementary Table 1: Plasma IGF-1 by baseline characteristics of patients in training (N = 310), first validation (N = 155), and second validation (N=100) cohorts

Patient characteristic	Parameter	Training cohort N=310 (%)			First validation cohort N=155 (%)*			Second validation cohort N=100 (%)		
		Plasma IGF-1 Level (ng/mL) Mean ± SD		P value	Plasma IGF-1 Level (ng/mL) Mean ± SD		P value	Plasma IGF-1 Level (ng/mL) Mean ± SD		P value
**Absolut values**		54.02	38.05		67.5	93		45.9	19.3	
**Age (years)**	≤60	58.1	44.4	0.6	64.4	89.8	0.7	46.1	19.9	0.9
	>60	50.9	32.1		69.8	95.8		45.6	18.4	
**Sex**	Male	51.8	35.7	0.2	61.3	90.9	0.2	44.8	19.4	0.2
	Female	59.2	42.8		84.1	97.7		51.6	18.1	
**Viral hepatitis**	HCV, HBV, or both	49.9	36.6	0.02	70.5	84.9	0.7	45.9	19.3	
	None	57.4	39		64.5	100.8		-----	-----	
**Serum α-FP (ng/mL)**	<400	56.9	36.1	0.002	73.6	100.9	0.3	49.4	20	0.09
	≥400	49.3	42.1		56.7	76.7		42.8	18.2	

**Tumor differentiation**	Well	56.6	37.9	0.07	72.6	98.01	0.9	36.7	24.6	0.3
	Moderate	55.8	46.9		75.7	87.02		43.1	18.5	
	Poor	47.4	38.1		67.1	101.01		34.4	22.7	
**Tumor nodularity**	Uninodular	63.1	41.7	< .001	87.6	103	0.7	45.8	19.5	<.0001
	Multinodular	49.4	38.5		76.6	99.1		24.1	8.4	
**% of liver involvement**	≤50%	59.9	41.1	< .001	71.5	91	0.4	46.8	19.3	0.002
	>50%	42	25.8		57.8	99.4		33.8	16.9	
**Vascular invasion**	Yes	42.7	32	< .001	59.6	95.5	0.2	23.6	10.2	<.0001
	No	58.7	39.4		88.6	100.7		45.8	19.2	
**Lymph node metastasis**	Yes	53.7	42.8	0.1	62.3	94.2	0.4	41.4	18.9	0.03
	No	54.8	34.6		74.6	91.5		50	18.9	
**Extrahepatic metastasis**	Yes	54.5	39.5	0.7	58.9	97.6	0.2	45.7	19.1	0.9
	No	54.2	37.7		77.9	86.6		46	19.5	
**ALT (U/L)**	≤40	61	44.3	0.02	75.1	91.3	0.4	49.3	19.1	0.2
	>40	48.7	31.5		61.6	94.4		43.7	19.2	
**AST (U/L)**	≤45	70	43.4	<.001	75.1	91.3	0.005	49.5	16.7	0.4
	>45	46.9	33.2		61.6	94.4		44.5	19.7	
**Platelet (mcl)**	≤100	43.9	36.6	0.04	44.3	64.1	0.1	40.9	17.4	<.0001
	>100	54.5	38.8		73.5	98.5		55.2	19.8	

**Na (mEq/L)**	≤130	32	23.2	0.02	78.6	131	0.8	31.7	10.9	<.0001
	>130	54.8	38.4		66.8	92.3		50.3	19.8	
**Cirrhosis**	No	65.4	40.9	<.001	99.7	115.3	0.001	50.1	15.7	0.03
	Yes	65.4	40.9		48.5	68.4		39.3	19.8	
**CTP score**	A	59.5	39	<.001	76.1	98.2	0.05	54.1	17.8	0.001
	B	41.2	32.4		33.5	55.7		46.7	18.1	
	C	34.1	33.1		7.9	12.7		34.7	17.5	
**BCLC stage**	0	58.2	29.7	0.3	120	79.2	0.6	----	----	0.001
	A	65.2	48.7		58	63		75	18.9	
	B	50.8	26.2		77.8	79.9		52.3	15.8	
	C	54.5	39.6		68.1	98.8		50.2	18.6	
	D	43.8	30.6		7.9	12.7		35.1	17	
